# Pharmacological Assessment of *Calamus guruba* Methanolic Extract: Multitarget Effects on Diabetes, Inflammation, Anxiety, and Depression

**DOI:** 10.1002/fsn3.72168

**Published:** 2026-07-30

**Authors:** Mohi Uddin, Tania Akter Shorna, Khurshida Jahan Suma, Md. Ekramul Haque Ekram, Masud Rana, Md. Jahirul Islam Mamun

**Affiliations:** ^1^ Department of Pharmacy, Faculty of Biological Sciences University of Chittagong Chittagong Bangladesh; ^2^ Department of Genetic Engineering and Biotechnology, Faculty of Life and Earth Science Jagannath University Dhaka Bangladesh

**Keywords:** alpha‐amylase, antidiabetic, antidepressant, anti‐inflammatory, anxiolytic, *Calamus guruba*

## Abstract

*Calamus guruba* Buch.‐Ham. (Family: Arecaceae) is a traditionally valued medicinal plant recognized for its wide range of therapeutic applications. This study was designed to evaluate the antidiabetic, anti‐inflammatory, anxiolytic, and antidepressant properties of the methanolic extract of *C. guruba* (MECG). Phytochemical screening of the extract was performed according to standard protocols. The antidiabetic potential was assessed using an alpha‐amylase inhibition assay. At the same time, anti‐inflammatory effects were examined using both in vitro (protein denaturation) and in vivo (carrageenan‐ and formalin‐induced paw edema) models. Anxiolytic activity was evaluated using the elevated plus maze (EPM) and hole board test (HBT), and antidepressant effects were determined through the tail suspension test (TST). MECG demonstrated potent antidiabetic activity, with an IC_50_ value of 48.69 μg/mL. The extract also showed marked anti‐inflammatory effects in all tested models. MECG showed significant anxiolytic activity (*p* < 0.001) in both the EPM and HBT at a dose of 400 mg/kg. Moreover, the same dose significantly decreased immobility time in the TST compared to the control group, indicating robust antidepressant‐like effects. Collectively, these findings suggest that MECG holds promise as a natural therapeutic agent for the management of diabetes, inflammation, anxiety, and depression.

## Graphical abstract

*Calamus guruba* Buch.‐Ham. (family Arecaceae) is a highly regarded traditional medicinal plant known for its diverse therapeutic benefits. This research aimed to investigate the antidiabetic, anti‐inflammatory, anxiolytic, and antidepressant effects of the methanolic extract of *C. guruba* (MECG). Standard protocols were utilized for the phytochemical screening of the extract. The antidiabetic efficacy was evaluated using an alpha‐amylase inhibition assay, while the anti‐inflammatory activity was assessed in vitro (protein denaturation) and in vivo (carrageenan‐ and formalin‐induced paw edema).

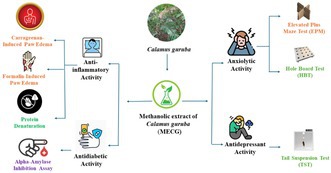

## 1 Introduction

Hyperglycemia is a hallmark of diabetes mellitus, a chronic metabolic illness. This disrupts the metabolism of proteins, lipids, and carbs and is due to insufficient insulin production by the pancreas or the body not responding to it (Nair et al. 2013). Ninety percent of the high prevalence of diabetes in urban communities is caused by type II diabetes (Wickramaratne et al. 2016). Its complications are extensive, encompassing macrovascular disorders like stroke and heart disease, as well as microvascular issues such as nephropathy and retinopathy (Forouhi and Wareham 2010). Traditional treatment focuses solely on restoring pancreatic islet function and managing blood glucose levels, failing to address diabetes‐related complications (Kania et al. 2011). Moreover, metformin, pioglitazone, and other oral antihyperglycemic medications, as well as insulin injections, have adverse effects (Fuangchan et al. 2011).

Inflammation is an immunological response to harmful stimuli of living tissue, such as infections, damaged cells, or irritants, and it manifests as pain, redness, and swelling (Chen et al. 2017). Although NSAIDs are standard treatments for pain and inflammation, they can cause adverse digestive effects, including indigestion, ulcers, and severe bleeding (Al Mahmud et al. 2016). Anxiety as well as depression are the two most prevalent mental illnesses, and when combined, they are often thought to be a part of the more widespread internalizing disorder (Kalin 2020). The most common mental disorder in people is anxiety, which is characterized by an unpleasant feeling of unknowable fear or anxiety (Kessler et al. 2005). According to figures from the World Health Organization (WHO), 260 million people worldwide suffer from anxiety (Leiva Nina et al. 2022). Besides, suicide can result from depression (Hossain et al. 2025). To treat anxiety, despite being commonly used, benzodiazepines (BZDs) such as diazepam, lorazepam, midazolam, and alprazolam are less effective over time, have mild to severe side effects, and are mostly abused (Hozack et al. 2022).

To treat human illnesses, bioactive chemicals from medicinal plants have long been used, providing therapeutic advantages with frequently less adverse effects than synthetic medications (Nahar et al. 2025; Hasan et al. 2025). They are the source of more than 30% of contemporary medications, carrying on a long history of natural cures (Hassan et al. 2013). Medicinal plants are now globally significant, with their proven therapeutic benefits making them a key resource for both research and alternative medicine (Zahra et al. 2019). According to estimates from the WHO, more than 80% of people on the planet still rely on traditional plant‐based medicines to meet their basic medical needs (Proestos 2020). Additionally, one of the most plentiful sources of pharmacologically active compounds for the manufacturing of novel drugs is medicinal plants (Mohammad, Md. Anisul, et al. 2025; Jyoti et al. 2020).

The climbing rattan palm, *Calamus guruba* Buch.‐Ham. ex Mart., belongs to the Arecaceae family and is native to northeastern India, Bangladesh, and neighboring regions of Southeast Asia (Meena et al. 2018). *C. guruba* leaves have flattened spines on the rachis and sheath, which are characteristic of many *Calamus* rattans, and sheathing leaf bases with brownish hairs (Evans et al. 2002). *C. erectus* methanol extract has a strong antihyperglycemic effect, according to research. The antipyretic, antinociceptive, gastrointestinal, neuropharmacological, and antidiarrheal properties of *C. tenuis* leaf extract were shown in another study (Sultana et al. 2022). *Calamus* leaves also exhibited anti‐inflammatory, antioxidant, and hepatoprotective activities (Anwar et al. 2023). Research on the phytochemistry and pharmacology of *Calamus* species, including *C. guruba*, reveals that leaf extracts contain a diverse range of bioactive components and exhibit various biological activities.

Along with other *Calamus* species, comparative leaf‐extract investigations reveal that *C. guruba* contains tannins, alkaloids, flavonoids, phenolic compounds, saponins, phytosterols, quinones, and terpenoids. Although most research to date has been preliminary and focused on cross‐species comparisons, these results indicate that *C. guruba* leaves are an encouraging source of secondary metabolites that may be useful in medicine (Sultana et al. 2022; Das et al. 2017).

Despite the limited and fragmented research on *C. guruba*, this study presents the first comprehensive, multitarget investigation of its pharmacological potential. By integrating in vitro assays with validated in vivo models, we systematically evaluate the extract's efficacy against diabetes, inflammation, and neurological disorders—key interconnected pathological conditions. This holistic approach not only addresses a critical knowledge gap but also establishes a scientific foundation for the plant's translational relevance in the development of evidence‐based phytomedicines.

## 2 Materials and Methods

### 2.1 Chemicals

All chemicals and reagents used in this study were of high analytical grade and procured from reputable commercial suppliers to ensure experimental accuracy, reproducibility, and consistency. Key solvents and reagents were sourced from established manufacturers, including Merck (India) and Sigma‐Aldrich (USA). Reference drugs—including fluoxetine, diclofenac sodium, and diazepam—were generously provided by Square Pharmaceuticals Limited (Bangladesh). Diethyl ether was supplied by Beximco Pharmaceuticals Limited. All other reagents and chemicals, of reagent grade, were sourced from the Department of Pharmacy, University of Chittagong.

### 2.2 Collection and Identification of the Plant

*Calamus guruba* leaves were collected in August 2024 from the Hathazari campus of the University of Chittagong, Chattogram, Bangladesh. The plant material was authenticated as *Calamus guruba* by Dr. Shaikh Bokhtear Uddin, Professor and Taxonomist, Department of Botany, University of Chittagong. A voucher specimen (No. CGL‐12/24) has been deposited in the Department of Pharmacy, University of Chittagong for future reference.

### 2.3 Extraction

After being gathered, the leaves were cut into small pieces, rinsed with fresh water, and allowed to air‐dry for 14 days. The high‐capacity grinder was used to grind the dry leaf into a coarse powder. To extract the material, 950 g of leaf was soaked in 4 L of methanol in a capped round‐bottom flask for 15 days, with occasional shaking. The entire combination was then coarse‐filtered using Whatman filter paper and a piece of white, sterilized cotton fabric. A rotary evaporator (Bibby RE‐200, Sterilin Ltd., UK) set to 4 rpm and 65°C was used to make the extract. It produced a dark‐green, sticky concentrate. The gummy concentrate, sometimes referred to as the crude extract or methanolic extract, was kept in a refrigerator at 4°C for subsequent use (Mamun, Rasel, et al. 2025; Mamun, Akter, et al. 2026). The yield percentage was 11.35%.

### 2.4 Experimental Animals and Ethical Statement

Swiss albino mice (22–30 g) of both sexes (4–5 weeks old) were obtained from the Bangladesh Council of Scientific and Industrial Research (BCSIR), Chattogram, Bangladesh. The animals were acclimatized and housed under controlled environmental conditions: temperature 25°C ± 1°C, relative humidity 60% ± 5%, and a 12‐h light/dark cycle. Prior to each experiment, mice were fasted for 12 h with ad libitum access to water to minimize variability in pharmacokinetic and behavioral responses (Mesu et al. 2026). All experimental procedures were approved by the Animal Ethics Review Board (AERB) of the University of Chittagong (Approval No. AERB‐FBSCU‐2024‐67). Physically ill or behaviorally abnormal mice were excluded from the study to ensure the reliability and validity of the experimental results. Only healthy animals, as confirmed by general physical examination and acclimatization observation, were included in the experimental groups. At the conclusion of the experiments, animals were humanely euthanized using diethyl ether anesthesia. The ARRIVE standards were followed throughout the investigation to ensure ethical conduct and transparent documentation of animal research.

### 2.5 Experiment Design

Mice were randomly divided into four groups of five animals each. Each group—including the control, standard drug, and two test dose groups of the *C. guruba* methanolic extract (MECG) —comprised a mixed‐sex cohort of five Swiss albino mice (both male and female) to ensure gender‐balanced representation across experimental conditions. The test groups were given MECG by ingestion at 200 and 400 mg/kg, b.w., respectively, while the control group received 1% Tween 80 in water (10 mL/kg, b.w.). Diclofenac sodium (10 mg/kg) (10 mg/kg b.w., i.p.) was utilized for the carrageenan‐induced paw edema test and the formalin‐induced paw edema test. The conventional drugs diazepam (1 mg/kg, p.o. b.w.) and fluoxetine (1 mg/kg, p.o. b.w.) were administered orally for the elevated plus maze, hole‐board, and tail suspension tests (Tareq et al. 2020).

### 2.6 Phytochemical Screening

The freshly prepared crude extract was analyzed for the main types of bioactive secondary metabolites. Using established chemical screening protocols, the presence of tannins, phenolic compounds, alkaloids, flavonoids, phytosterols, saponins, quinones, and other phytoconstituents was confirmed through characteristic color changes and precipitation reactions (Ghani 2003; Shaikh and Patil 2020).

### 2.7 Acute Toxicity Assay

Following the recommendations of the Organization for Economic Co‐operation and Development (OECD), acute toxicity tests were carried out. Both male and female Swiss albino mice weighing 22–30 g were randomly assigned to five groups (*n* = 5 per group). Before treatment, baseline behavioral parameters were assessed and documented for each animal. Every mouse had unrestricted access to water despite fasting all night. One group was given normal saline (10 mL/kg, i.p.) as the control. In contrast, the other three groups were administered the plant extract (MECG) intraperitoneally at doses of 1, 3, and 5 g/kg, respectively. Following administration, animals were closely monitored for behavioral changes and physiological responses, including alertness, convulsions, grooming, lacrimation, hyperactivity, sweating, urination, righting reflex, pain response, corneal reflex, grip strength, touch response, and mortality, at predetermined time intervals: 0.5, 1, 2, 4, 6, 12, 24, and 48 h post‐dosing (Lalitha et al. 2012; Mamun, Apu, et al. 2026).

### 2.8 Antidiabetic Activity

#### 2.8.1 In Vitro Alpha‐Amylase Enzyme Assay

The positive control in this evaluation was acarbose. In a test tube, 1 mL of extract MECG and 1 mL of alpha‐amylase (Square Pharmaceuticals) were incubated at 37°C for 10 min. Each tube received 1 mL of 1% (v/v) starch solution after preincubation, and the tubes were thereafter incubated for 15 min at 37°C. The mixture was heated in a boiling water bath for 5 min, cooled to room temperature, and then diluted with 2 mL of DNSA reagent after the reaction was stopped. Both the methanolic extract of *C. guruba* (MECG) and the standard drug acarbose were tested at concentrations of 10, 20, 40, 80, and 160 μg/mL to evaluate their concentration‐dependent effects in in vitro assays. The absorbance was then calculated at 546 nm using a Shimadzu spectrophotometer. The control procedure, which reflected 100% enzyme activity, did not include any plant extract. Acarbose and the extract were given at varying dosages (10–160 μg/mL) (Quazi et al. 2022). Alpha‐amylase inhibition percentage by extract and acarbose can be computed:

$$\%\text{Inhibition}=\frac{\text{Absorbance of Control}-\text{Absorbance of Extract}}{\text{Absorbance of Control}}\times 100$$

### 2.9 Anti‐Inflammatory Activity

#### 2.9.1 Egg Albumin Denaturation Assay

The method was followed with modifications as described in a previously published method (Aidoo et al. 2021). 2.0 mL of MECG at different doses (62.5, 125, 250, 500, and 1000 μg/mL), 2.8 mL of buffered phosphate saline (PBS, pH 6.4), and 0.2 mL of fresh egg albumin were combined to form the reaction mixture (5 mL). 2.0 mL of diclofenac sodium at various concentrations (62.5, 125, 250, 500, and 1000 μg/mL), 0.2 mL of fresh egg albumin, and 2.8 mL of PBS (pH 6.4) made up the positive control. PBS with 2.0 mL of distilled water and an equal quantity of egg albumin were present in the negative control samples. To induce denaturation, the mixture was heated to 70°C for 5 min after incubation at 37°C ± 2°C for 15 min. After cooling, a UVmini‐1240 spectrophotometer (Shimadzu) was used to measure absorbance at 660 nm with the vehicle as the blank. Three duplicate tests were run. The % inhibition of protein denaturation was determined through the formula:

$$\%\text{Inhibition}=\frac{\text{Absorbance of Control}-\text{Absorbance of Extract}}{\text{Absorbance of Control}}\times 100$$

#### 2.9.2 Carrageenan‐Induced Paw Edema Assay

In accordance with Kumar et al., the carrageenan‐induced paw edema test was used to assess the anti‐inflammatory properties of the methanolic extract of *C. guruba* (MECG) (Kumar et al. 2012). In order to cause acute inflammation, 0.1 mL of 1% carrageenan solution was subcutaneously injected into each mouse's left hind paw's plantar region. A digital plethysmometer (IITC Life Science, USA) was used to measure paw volume before the baseline carrageenan injection and at 1, 2, 3, and 4 h after the injection. Mice were randomly segmented into four groups (*n* = 5):

- Group I (Vehicle control): 1% Tween 80;
- Group II (Positive control): received diclofenac sodium (10 mg/kg, p.o.);
- Group III: treated with MECG (200 mg/kg, p.o.);
- Group IV: treated with MECG (400 mg/kg, p.o.).

Test agents were administered orally 30 min after carrageenan injection. The percentage inhibition of edema was calculated using the formula:

$$\%\text{Inihibition}=\frac{\mathrm{Vc}-\mathrm{Vt}}{\mathrm{Vc}}\times 100$$

where Vc = mean paw volume in the control group and Vt = mean paw volume in the treated groups at each time point.

#### 2.9.3 Formalin‐Induced Paw Edema

Based on a previously published method (Soyocak et al. 2019), a sub‐plantar injection of 0.1 mL of 5% formalin was given to mice in each group to induce edema in the left hind paw. The right hind paw was maintained as a negative control. The anti‐inflammatory activity of the treatment groups (MECG 200 and 400 mg/kg) and the control group was compared using the plethysmograph technique. Each group's left and right paw volumes were measured at zero time (normal paw volume) as well as 1, 2, 3, and 4 h following the induction of inflammation. The % inhibition of edema was measured as follows:

$$\%\text{Edema Inhibition}=\frac{\left(\mathrm{VL}-\mathrm{VR}\right)\text{control}-\left(\mathrm{VL}-\mathrm{VR}\right)\text{treated}}{\left(\mathrm{VL}-\mathrm{VR}\right)\text{control}}\times 100$$

where VL stands for the left paw displacement volume mean, and VR for the right paw displacement volume mean.

### 2.10 Anxiolytic Activity

#### 2.10.1 Elevated Plus Maze Test

This widely recognized test was used to evaluate rodents' anxiolytic qualities (Emon et al. 2020). The distance between the floor and the raised plus‐maze was 50 cm. The five animals were divided into three groups: test, positive control, and negative control. Following treatment with either diazepam (1 mg/kg), the test extracts (200 and 400 mg/kg), or the vehicle, each mouse was placed in the center of the labyrinth and guided toward the closing arms. For 5 min at 0, 30, 60, 90, and 120 min, the total number of open and closed arm entries, as well as the amount of time spent in each, were noted. A sound‐attenuated environment was used to monitor the entire operation.

#### 2.10.2 Hole Board Test

The hole board device was a 40 × 40 × 25 cm wooden chamber with 16 uniformly placed holes (3 cm in diameter). The device was constructed with a platform 25 cm above the ground so the mice could see through the holes. The mice were given diazepam (1 mg/kg, oral), the vehicle (10 mL/kg, oral), and MECG (200 and 400 mg/kg, oral) 30 min prior to the test commencing. The amount and duration of each animal's head poking were noted during the 5‐min observation session (Emon et al. 2022).

### 2.11 Antidepressant Activity

#### 2.11.1 Tail Suspension Test (TST)

The rodents used in this test were divided into four groups, each comprising five mice. The test groups were designed according to Section 2.5. Thirty minutes after the animals were given the drug, they were suspended 50 cm above the ground with sticky tape that was put nearly 1 cm from the tip of their tails. The animals were immobile for 6 min at that time. The mice were supposed to be inactive when they hung silently or did not move (Mohammad, Chowdhury, et al. 2025; Emon et al. 2021).

### 2.12 Statistical Analysis

For each concentration, the findings are presented as mean ± SEM from triplicate measurements. Analysis of variance (ANOVA) and Dunnett's *t*‐test were used for statistical analysis. GraphPad Prism software version 8.0 (GraphPad Software Inc., San Diego, CA, USA) and MS Excel 2024 were used for creating the graphs (Mamun, Mizan, et al. 2026). *p*‐Values of less than 0.05, 0.01, and 0.001 (*p < 0.05, **p < 0.01, ***p < 0.001) were considered statistically significant when compared to the control.

## 3 Results

### 3.1 Phytochemical Screening

The phytochemical screening results of the methanolic extract of *C. guruba* are detailed in Table 1.

**TABLE 1:** Phytochemical screening of the methanolic extract of *C. guruba*.

Secondary metabolite	Name of the test	Observation	Result
Alkaloids	Wagner's test	Brown or deep brown precipitate	++
	Dragendroff's test	A reddish‐brown precipitate	++
	Mayer's test	A creamy white/yellow precipitate	−−
	Tannic acid test	A buff‐colored precipitate	++
	Iodine test	A blue color	−−
Reducing sugar	Fehling's test	A red precipitate	−−
Glycosides	10% NaOH test	A brick red precipitate	−−
	Aqueous NaOH test	A yellow color	−−
	Concentrate H_2_SO_4_ test	A brown ring	−−
Cardiac glycosides	Keller‐Killani test	A blue‐colored solution	−−
Flavonoids	Alkaline reagent test	An intense yellow color becomes colorless	++
	Ammonia test	A yellow color	−−
Phlobatannins	NaHCO_3_ test	A honeycomb‐like froth	−−
	Olive oil test	An appearance of foam	−−
Terpenoids	Chloroform + H_2_SO_4_ test	A gray‐colored solution	++
Triterpenoids	Salkowski's test	A golden yellow layer (at the bottom)	++
Phytosterols	Salkowski's test	A red color (in the lower layer)	++
Phenol	Lead acetate test	A white precipitate	++
	Potassium dichromate test	A dark color	++
Quinones	Alcoholic KOH test	Red to blue color	++
Tannins	10% NaOH test	Formation of emulsion	++
Carboxylic acid	Effervescence test	Appearance of effervescence	++
Saponin	Foam test	Persistent foam for 10 min	++
	NaHCO_3_ test	Stable honeycomb‐like froth	++
	Olive oil test	Appearance of foam	++

*Note:* “++” stands for the presence and “−−” indicates the absence of secondary metabolites.

### 3.2 Acute Oral Toxicity Study

Animals given oral doses of 1000, 2000, 3000, or 4000 mg/kg of MECG did not exhibit any fatal side effects or behavioral toxicity indicators, such as defecation, urination, lacrimation, salivation, piloerection, aggression, hyperactivity, convulsions, tremors, or twitching. With an estimated LD_50_ surpassing 4000 mg/kg, MECG was therefore considered safe even at the highest tested dose of 4000 mg/kg. These results led to the selection of extract doses (200 and 400 mg/kg) for further in vivo investigations, which were deemed safe and non‐toxic.

### 3.3 In Vitro Antidiabetic Activity

#### 3.3.1 Alpha‐Amylase Inhibitory Assay

**FIGURE 1:**
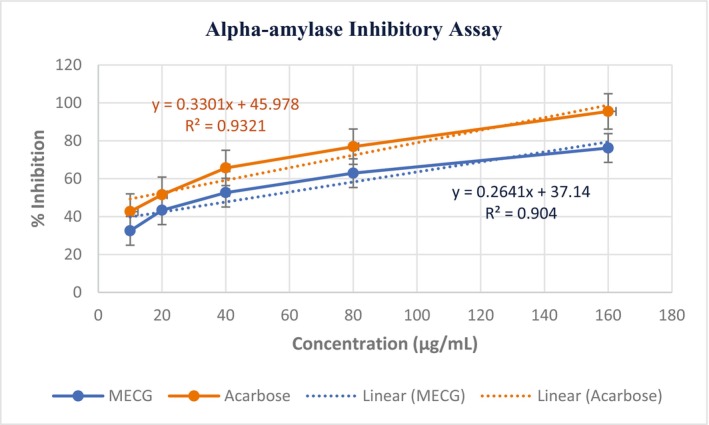
Graphical representation of antidiabetic activity of MECG through alpha‐amylase assay. The test was done in triplicate.

The acarbose standard and the MECG extract both inhibited α‐amylase in a concentration‐dependent manner, with IC_50_ values of 12.18 and 48.69 μg/mL, respectively (Figure 1). Given its lower IC_50_, the MECG extract showed more potent inhibitory effects (76.18% ± 0.1%), suggesting alpha‐amylase inhibition similar to that of acarbose (95.47% ± 2.44%).

### 3.4 Anti‐Inflammatory Activity

#### 3.4.1 Protein Denaturation Assay

**FIGURE 2:**
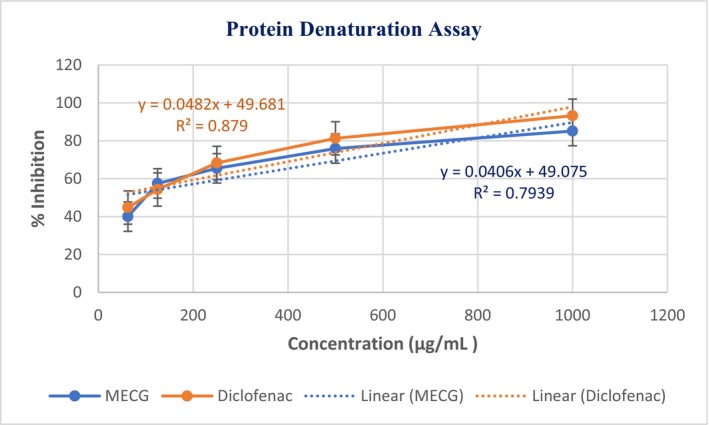
Anti‐inflammatory activity by calculating % inhibition using protein denaturation assay. The test was done in triplicate.

MECG's anti‐inflammatory properties in vitro were evaluated by its capacity to prevent protein denaturation. The mean inhibition percentages of MECG at 1000, 500, 250, 125, and 62.5 μg/mL were 85.12% ± 0.58%, 75.92% ± 0.33%, 65.45% ± 0.44%, 57.5% ± 1.23%, and 40% ± 0.46%, respectively, indicating a concentration‐dependent effect. Despite MECG's significant anti‐inflammatory potential, traditional diclofenac sodium showed greater efficacy, with a maximum inhibition of 93.21% ± 1.58% at 1000 μg/mL and an IC_50_ of 6.62 μg/mL. The IC_50_ value for the extract was 22.78 μg/mL (Figure 2).

#### 3.4.2 Carrageenan‐Induced Paw Edema Test

**FIGURE 3:**
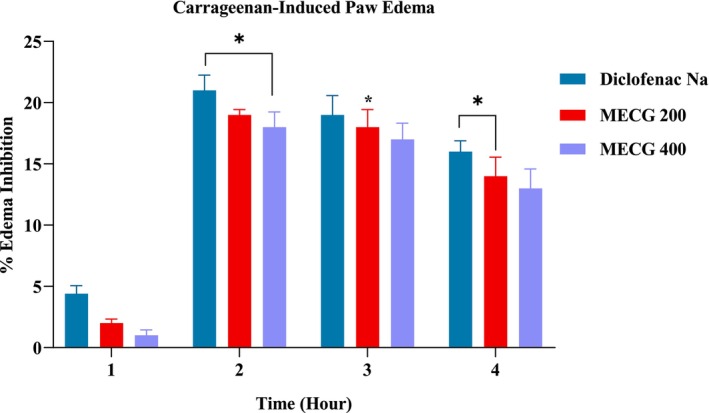
Measurement of %edema inhibition of mice through the MECG treatment in the Carregenan‐induced test. Each value represents the mean ± SEM (*n* = 5). One‐way ANOVA followed by Dunnett's *t*‐test. *p 〈 0.05, compared with control.

In our research, mice that received a subcutaneous injection of carrageenan developed edema, increasing paw size, and suggesting acute paw inflammation. The thickness of the paws in several mouse groups after receiving MECG and diclofenac sodium is displayed in Table 2. At 4 h, MECG and diclofenac sodium were more effective than placebo in inhibiting the edematous response (*p* < 0.05). Edema inhibition was significant at 2 h after the extract treatment (Figure 3).

**TABLE 2:** Effects of anti‐inflammatory assay by carrageenan‐induced paw edema test on the treated mice.

Treatment	Pre‐injection paw circumference (mm)	Post‐injection paw circumference (mm)			
		1 h	2 h	3 h	4 h
Control	0.95 ± 0.163	1.05 ± 0.09	1.34 ± 0.09	1.35 ± 0.10	1.31 ± 0.03
Diclofenac Na	1.0 ± 0.151	1.00 ± 0.14	1.07 ± 0.07*	1.1 ± 0.14	1.11 ± 0.09*
MECG 200	1.05 ± 0.22	1.038 ± 0.09	1.09 ± 0.09*	1.12 ± 0.08*	1.13 ± 0.06*
MECG 400	1.05 ± 0.11	1.04 ± 0.11	1.1 ± 0.08*	1.12 ± 0.11	1.14 ± 0.105

*Note:* Each value represents the mean ± SEM (*n* = 5). One‐way ANOVA followed by Dunnett's *t*‐test. *p < 0.05 compared with control.

#### 3.4.3 Formalin‐Induced Paw Edema Test

**FIGURE 4:**
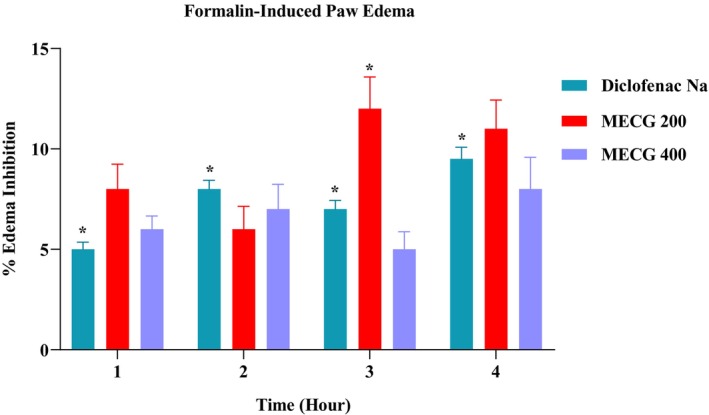
Measurement of %edema inhibition of mice through the MECG treatment. Each value represents the mean ± SEM (*n* = 5). One‐way ANOVA followed by Dunnett's *t*‐test. *p 〈 0.05, compared with control.

According to Table 3, the in vivo anti‐inflammatory effect was evident, as the paw thickness of the Formalin control group increased significantly at 5 h. It then steadily decreased at each hourly interval (1–4 h). The MECG significantly reduced paw thickness at both dosages (200 and 400 mg/kg) at the third and fourth hours. In contrast, the 200 mg/kg dose showed significant effectiveness against edematous response during the same time period. The 400‐mg/kg dose exhibits vigorous activity from the second to the fourth hour, almost identical to that of the regular medication (Figure 4).

**TABLE 3:** Effects of anti‐inflammatory assay by formalin‐induced paw edema test on the treated mice.

Treatment	Pre‐injection paw circumference (mm)	Post‐injection paw circumference (mm)			
		1 h	2 h	3 h	4 h
Control	1.46 ± 0.248	1.68 ± 0.259	1.78 ± 0.057	1.81 ± 0.08	1.9 ± 0.27
Diclofenac Na	1.48 ± 0.288	1.61 ± 0.29*	1.65 ± 0.25*	1.7 ± 0.24*	1.67 ± 0.25*
MECG 200	1.61 ± 0.332	1.55 ± 0.17	1.69 ± 0.27	1.6 ± 0.09*	1.7 ± 0.141
MECG 400	1.62 ± 0.36	1.59 ± 0.25	1.67 ± 0.28	1.72 ± 0.08	1.75 ± 0.105

*Note:* Each value represents the mean ± SEM (*n* = 5). One‐way ANOVA followed by Dunnett's *t*‐test. *p < 0.05 compared with control.

### 3.5 Anxiolytic Activity

#### 3.5.1 Elevated Plus Maze Test

**FIGURE 5:**
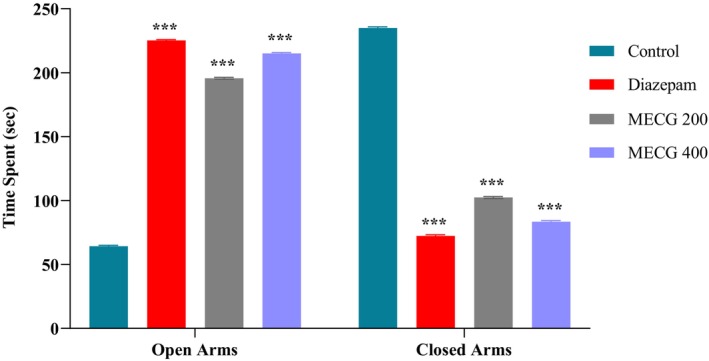
Screening of anxiolytic activity of MECG by elevated plus maze test. Values are Mean ± SEM (*n* = 5); ***p 〈 0.001 as compared to vehicle control (one‐way ANOVA followed by Dunnett's test).

MECG at 200 and 400 mg/kg significantly increased time spent in open arms, indicating anxiolytic activity, as measured by the Elevated Plus Maze (EPM) test (Table 4 and Figure 5). The open arm time increased to 195 ± 0.75 s (*p* < 0.01) at 200 mg/kg. In contrast, the closed arm time decreased to 48 ± 0.79 s compared to the control (8.1 ± 0.55 s). At 400 mg/kg, closed arm time dropped to 35 ± 0.89 s, but open arm time increased to 215 ± 0.68 s (*p* < 0.001). Compared to 8 in controls, transition frequencies to open arms were 8 and 10. The most significant effect was observed with diazepam (1 mg/kg) (225 ± 0.69 s in the open arm). These findings support the dose‐dependent anxiolytic potential of MECG.

**TABLE 4:** Effects of the extract on the treated mice in the elevated plus maze test.

Group	Time spent in open arms	No. of entries in open arm	Time spent in closed arms	No. of entries in closed arm
Control	64.33 ± 0.71	8 ± 0.55	235 ± 0.89	17 ± 0.73
Diazepam	225.22 ± 0.69***	16 ± 0.85***	72.35 ± 0.82***	8 ± 0.71***
MECG 200	195.56 ± 0.75***	8.4 ± 0.89	102.37 ± 0.79***	6 ± 0.71***
MECG 400	215.12 ± 0.68***	10 ± 0.71	83.35 ± 0.89***	4.6 ± 0.89***

*Note:* Values are Mean ± SEM (*n* = 5); ***p < 0.001 as compared to vehicle control (one‐way ANOVA followed by Dunnett's test).

#### 3.5.2 Hole Board Test

**FIGURE 6:**
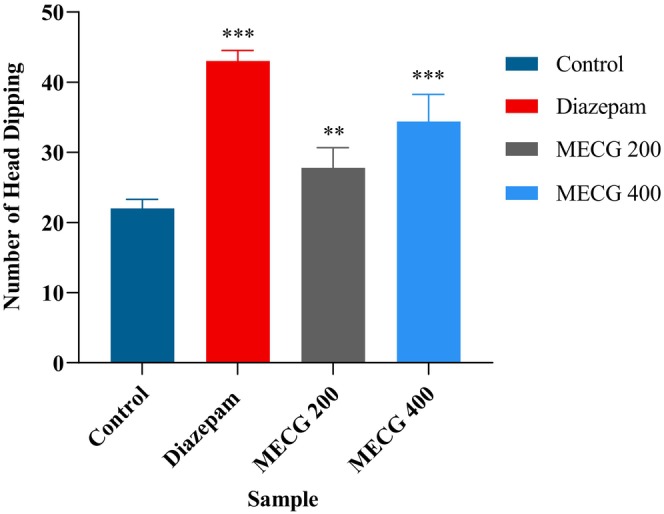
Screening of the anxiolytic activity of MECG by hole board test. Values are Mean ± SEM (*n* = 5);**p 〈 0.01, and ***p 〈 0.001 as compared to vehicle control (one‐way ANOVA followed by Dunnett's test).

The methanolic extract of *C. guruba* increased head‐dipping behavior in the hole‐board test, indicative of increased exploratory activity and decreased anxiety, as shown in Table 5. MECG generated 27.8 ± 2.87 and 34.4 ± 3.88 head dips at 200 and 400 mg/kg, respectively. Interestingly, 400 mg/kg of MECG had a substantial anxiolytic effect (*p* < 0.001) that was comparable to that of the standard medication diazepam. On the other hand, the 200 mg/kg dose had a less severe effect, suggesting a dose‐dependent reaction (*p* < 0.01) (Figure 6).

**TABLE 5:** Effects of the extract on the treated mice in the hole board test.

Animal group	Frequency of dipping					Mean ± SEM
	M‐1	M‐2	M‐3	M‐4	M‐5	
Control	23	18	25	20	24	22 ± 1.30
Diazepam	42	47	41	39	46	43 ± 1.52***
MECG 200	34	20	33	22	30	27.8 ± 2.87**
MECG 400	38	31	39	29	35	34.4 ± 3.88***

*Note:* Each value represents the mean ± SEM (*n* = 5). One‐way ANOVA followed by Dunnett's *t*‐test. **p < 0.01, ***p < 0.001 compared with control.

### 3.6 Antidepressant Activity

#### 3.6.1 Tail Suspension Method

**FIGURE 7:**
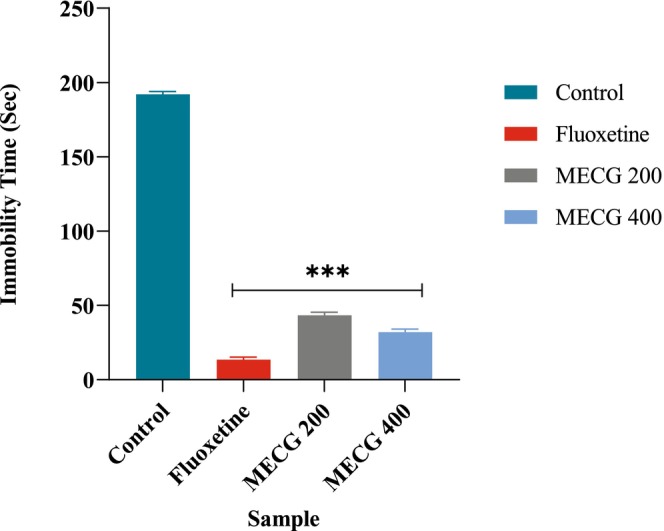
Screening of antidepressant activity of MECG through the tail suspension test. Values are Mean ± SEM (*n* = 5); ***p 〈 0.001 as compared to vehicle control (one‐way ANOVA followed by Dunnett's test).

The TST results (Figure 7) show that MECG exhibits dose‐dependent antidepressant effects. The control group had the highest immobility time (192 ± 1.79 s), indicating no activity. The standard drug Fluoxetine (1 mg/kg) significantly reduced immobility to 13.4 ± 1.86 s (*p* < 0.001). MECG at 200 mg/kg decreased immobility to 43.3 ± 2.11 s (*p* < 0.001), and 400 mg/kg further lowered it to 32 ± 2.00 s (*p* < 0.01), showing effects comparable to Fluoxetine. These findings highlight MECG's potential as a natural antidepressant.

## 4 Discussion

For generations, medicinal plants have been an integral part of traditional treatment, providing natural solutions for a variety of illnesses in many cultures (Mamun, Mizan, et al. 2025). In today's world, medicinal plants are recognized as game changers in mainstream disease treatment due to their diverse bioactive constituents. To assess the potential of new lead compounds, this study aims to explore the antidiabetic, anti‐inflammatory, anxiolytic, and antidepressant effects of the methanolic extract of *C. guruba*, informed by its traditional medicinal uses and documented phytochemical composition. Interest in plant‐based extracts and natural compounds for preventing and managing chronic diseases has been steadily increasing. These extracts typically harbor a wide array of bioactive molecules that exert health‐promoting effects through synergistic, multi‐target actions, simultaneously influencing or inhibiting multiple critical molecular pathways implicated in disease development (Khalid et al. 2023).

Phytochemical screening of the methanolic extract of *C. guruba* identified the presence of tannins, alkaloids, flavonoids, phenolic compounds, saponins, phytosterols, quinones, and terpenoids. The detection of these bioactive constituents suggests that the methanolic leaf extract of *C. guruba* may exhibit substantial biological activity. Among these, flavonoids are particularly noteworthy for their diverse pharmacological effects, with certain members of this class showing significant analgesic and anti‐inflammatory properties (Ramesh et al. 1998; Kim et al. 2004), mainly through their ability to inhibit key inflammatory mediators significantly (Middleton Jr 1998). Similarly, terpenoids exhibit considerable analgesic and anti‐inflammatory activities (Neukirch et al. 2005; Moody et al. 2006), which are often linked to their inhibition of phospholipase A_2_, an enzyme critical in the arachidonic acid metabolic pathway. Additionally, several alkaloids have been shown to exert anti‐inflammatory effects by interfering with the same arachidonic acid pathway, thereby suppressing the production of pro‐inflammatory metabolites (Ullah et al. 2014).

The α‐amylase inhibition assay demonstrated that the methanolic extract of *C. guruba* possesses notable inhibitory activity, with an IC_50_ value of 48.69 μg/mL, though less potent than acarbose (IC_50_ = 12.18 μg/mL), a standard antidiabetic drug commonly used in clinical settings. α‐Amylase inhibitors, often referred to as “starch blockers,” function by inhibiting the enzymatic digestion of starch and oligosaccharides into simpler sugars, such as maltose and maltotriose. This occurs primarily through the inhibition of hydrolysis at the 1,4‐glycosidic bonds, ultimately helping to lower postprandial blood glucose levels by reducing carbohydrate absorption in the gut (Dineshkumar et al. 2010). According to recent research, tannins are non‐specific, broad‐spectrum inhibitors of a variety of hydrolytic enzymes, such as invertase, lipases, α‐glucosidases, and α‐amylases (Elya et al. 2015). Phytochemical screening of the *C. guruba* methanolic extract confirmed the presence of tannins, suggesting they may contribute significantly to the observed α‐amylase inhibitory effect. This proposed role of tannins could be substantiated through further investigations, such as the isolation of tannin‐rich fractions from the extract and subsequent evaluation of their specific inhibitory activity against α‐amylase in purified form.

The methanolic extract of *C. guruba* (MECG) was investigated for its in vitro anti‐inflammatory properties using the heat‐induced denaturation of egg albumin as a model. In vivo denaturation of tissue proteins can result in the development of auto‐antigens in some types of autoimmune arthritis, and protein denaturation is a known cause of inflammatory and arthritic disorders (Opie 1962; Umapathy et al. 2010). The test samples, including MECG and the reference drug diclofenac sodium, demonstrated a dose‐dependent reduction in albumin denaturation, as evidenced by lower absorbance values compared to the control, indicating stabilization of the protein structure (Jagtap et al. 2011). Notably, MECG exhibited significant inhibitory activity, with an IC_50_ value comparable to that of diclofenac sodium, suggesting potent anti‐denaturation properties. Phytochemical analysis of MECG revealed the presence of alkaloids and phenolic compounds, particularly tannins. Polyphenols are well‐documented natural agents with diverse biological activities, including anti‐inflammatory effects (Bhattacharya 2011). The observed in vitro anti‐inflammatory activity of MECG is likely attributable to its rich polyphenolic content, potentially acting through synergistic interactions among multiple constituents rather than a single compound. It is also noteworthy that the ability to stabilize heat‐denatured albumin at physiological pH (6.2–6.5) is a recognized characteristic of several non‐steroidal anti‐inflammatory drugs (NSAIDs), further supporting the relevance of this assay (Chandra et al. 2012). Based on these preliminary findings, MECG demonstrates a significant in vitro anti‐inflammatory effect by inhibiting protein denaturation. However, more comprehensive studies, including bioactivity‐guided fractionation, mechanistic investigations, and in vivo validation, are warranted to identify the specific bioactive constituents and elucidate the underlying molecular pathways responsible for this activity.

An established experimental model for evaluating the anti‐inflammatory properties of substances and natural products is carrageenan‐induced paw edema (El‐Shenawy et al. 2002). Carrageenan causes a biphasic inflammatory response: mediators such as histamine, kinins, and serotonin are released during the first hour, and prostaglandin synthesis and lysosomal enzyme release are the main drivers of the second phase, which peaks between 2 and 4 h (Brooks and Day 1991). This latter phase is exceptionally responsive to conventional anti‐inflammatory drugs (Vinegar et al. 1969). In the present study, the methanolic extract of *C. guruba* (MECG) demonstrated significant inhibition of carrageenan‐induced acute inflammation at the 4‐h time point, with efficacy comparable to that of the standard diclofenac sodium. These findings suggest that the anti‐inflammatory activity of MECG may result from its ability to suppress the synthesis or activity of key inflammatory mediators in the second phase, particularly prostaglandins, proteases, and/or lysosomal enzymes.

The formalin‐induced paw edema model was used to evaluate the anti‐inflammatory properties of the methanolic extract of *C. guruba* (MECG). The results showed a time‐dependent decline in paw volume, indicating a progressive decrease in inflammation. The extract's capacity to inhibit the release or activity of key inflammatory mediators, including histamine, bradykinin, serotonin, and prostaglandins, is likely responsible for this anti‐edematous effect. Several bioactive components, including terpenoids, alkaloids, quinones, phytosterols, phenolic compounds, saponins, tannins, and flavonoids, were confirmed to be present in MECG by phytochemical analysis. These constituents have all been linked to anti‐inflammatory activity across a variety of experimental and clinical settings (Beck and Namdeo 2015). These constituents are known to exert their effects primarily by modulating the arachidonic acid cascade (Chi et al. 2001). Specifically, flavonoids can act as phospholipase A_2_ inhibitors and have also been reported to suppress tumor necrosis factor‐alpha (TNF‐α) in various inflammatory settings. Depending on their chemical structure, certain flavonoids can inhibit both cyclooxygenase (COX) and lipoxygenase (LOX) pathways, thereby blocking the synthesis of prostaglandins and leukotrienes (Jang et al. 2002). In the present study, MECG exhibited significant anti‐inflammatory activity at both tested doses. The observed effect is likely attributable to its high tannin and flavonoid content, which are known to interfere with prostaglandin synthesis and other inflammatory signaling pathways. Collectively, the results strongly suggest that *C. guruba* possesses notable anti‐inflammatory potential, primarily mediated by tannins and flavonoids, which suppress key enzymatic and signaling pathways involved in inflammation, particularly those linked to arachidonic acid metabolism.

A popular mouse model for examining anxiety‐like behaviors and the anxiolytic or anxiogenic potential of pharmaceutical drugs is the elevated plus maze (EPM) (Hogg 1996). It takes advantage of mice's innate dislike of wide, high areas, which usually leads to thigmotactic behavior, in which animals seek refuge behind walls or in enclosed arms (Carobrez and Bertoglio 2005). As described by Rodgers et al. (1997), the open‐arm versus closed‐arm paradigm in the EPM is particularly effective for identifying compounds that act via benzodiazepine‐like mechanisms. This approach was employed in the current study. Administration of the methanolic extract of *C. guruba* (MECG) at doses of 200 and 400 mg/kg significantly and dose‐dependently increased the time spent by mice on the open arms of the maze, a behavioral indicator of reduced anxiety. This result strongly suggests an anxiolytic‐like effect of MECG. Further support for this conclusion comes from the hole‐board test, which evaluates exploratory behavior and anxiety in response to a novel environment (Moreira et al. 2000). In this test, an increase in head‐dipping frequency is interpreted as a sign of anxiolysis (Chatterjee et al. 2011). Mice treated with MECG (400 mg/kg) showed a marked increase in head‐dipping behavior compared with the control group, further reinforcing the extract's anxiolytic‐like properties. Notably, the anxiolytic effect observed with MECG (400 mg/kg) was comparable in magnitude to that produced by diazepam, a standard benzodiazepine anxiolytic drug. Together, these findings from both the EPM and hole‐board tests provide convergent evidence that MECG possesses significant anxiolytic‐like activity.

Flavonoids have been increasingly recognized for their therapeutic potential in various neurological disorders, particularly depression and anxiety (Olubodun‐Obadun et al. 2023). Supporting this notion, a recent study by Chandrasekhar et al. (2018) demonstrated that a tannin‐rich extract from *Terminalia chebula* modulated key neurochemical and molecular pathways in a mouse model. Specifically, the extract significantly lowered serum cortisol levels while increasing the concentrations of monoamine neurotransmitters, namely serotonin (5‐hydroxytryptamine, 5‐HT), dopamine, and norepinephrine, in brain tissue. Furthermore, gene expression reveals upregulation of several mood‐regulating targets, including brain‐derived neurotrophic factor (BDNF), cAMP response element‐binding protein (CREB), GABA_A_ and 5‐HT_1A_ receptors, molecular markers closely associated with antidepressant and anxiolytic effects. In the present study, treatment with *C. guruba* extract (MECG) at a dose of 400 mg/kg similarly enhanced monoaminergic neurotransmission, likely by inhibiting monoamine oxidase A (MAO‐A) activity in the brain. This mechanism would reduce the breakdown of monoamine neurotransmitters, thereby increasing their synaptic availability. Given that phytochemical analysis confirmed the presence of flavonoids and tannin‐rich compounds in *C. guruba* leaves, it is plausible that these constituents underlie the observed neuropharmacological effects. The structural and functional similarities between the tannin‐ and flavonoid‐rich fractions of *C. guruba* and those of *T. chebula* suggest a comparable mode of action, potentially involving modulation of stress hormones, monoamine levels, and neurotrophic signaling pathways linked to mood regulation.

Animal models are indispensable in elucidating the pathophysiology of depression and in identifying novel therapeutic candidates with antidepressant potential (Bebeji and Yaro 2024). Among these, the Tail Suspension Test (TST), a well‐validated model of behavioral despair, effectively mirrors core symptoms of human depression (Steru et al. 1985; Porsolt et al. 1977). In this paradigm, mice initially struggle to escape the inescapable stress of being suspended by their tails but eventually exhibit increased immobility, interpreted as a state of “behavioral despair” (Yankelevitch‐Yahav et al. 2015). The duration of immobility during the 6‐min test session is widely accepted as the primary endpoint for assessing antidepressant‐like activity; a significant reduction in immobility time reflects enhanced escape‐oriented behavior. It is indicative of antidepressant efficacy (Abelaira et al. 2013). The TST is highly sensitive to clinically effective antidepressants, including monoamine oxidase inhibitors (MAOIs), selective serotonin reuptake inhibitors (SSRIs), and tricyclic antidepressants, all of which reliably decrease immobility and promote active coping behaviors (Steru et al. 1985; Porsolt et al. 1977). In the present study, the methanolic leaf extract of *C. guruba* (MECG), administered at all tested doses, produced a statistically significant (*p* < 0.001) reduction in immobility time compared to the control group, showing activity similar to the standard SSRI, fluoxetine (10 mg/kg). This finding strongly supports the antidepressant‐like activity of MECG in the TST model. Phytochemical screening of MECG revealed the presence of bioactive secondary metabolites, including tannins, saponins, and flavonoids, classes of compounds previously associated with neuroprotective and antidepressant effects in preclinical studies. These constituents may modulate monoaminergic neurotransmission, reduce oxidative stress, or influence neurotrophic signaling pathways, thereby contributing to the observed behavioral improvements (Bebeji and Yaro 2024). However, the specific phytochemical(s) responsible for the antidepressant effect, as well as the underlying molecular mechanisms, remain unclear. Consequently, while the current results provide compelling preliminary evidence for the antidepressant potential of *C. guruba*, further research, including bioactivity‐guided fractionation, isolation of active compounds, receptor‐binding assays, and neurochemical profiling, is warranted to identify the precise constituents and elucidate the mechanisms driving this promising pharmacological activity.

The phytochemical characterization remains preliminary, based on standard qualitative assays. However, the promising biological activities observed provide a strong rationale for future investigations using advanced analytical techniques such as HPLC, GC–MS, or LC–MS to isolate, identify, and validate the active constituents, thereby enabling a more robust structure–activity correlation and mechanistic understanding.

## 5 Conclusion

The methanolic extract of *Calamus guruba* (MECG) demonstrated significant in vitro antidiabetic and anti‐inflammatory properties and, in vivo, anxiolytic and antidepressant properties in validated experimental models, supporting its traditional medicinal use. These effects, particularly prominent at 400 mg/kg, suggest MECG as a promising candidate for developing natural therapeutics for diabetes, inflammation, anxiety, and depression. Consequently, these preliminary studies necessitate further research to isolate active compounds, thereby facilitating an understanding and clarification of the underlying mechanisms.

## Author Contributions

**Tania Akter Shorna:** conceptualization, methodology, writing – original draft, investigation, data curation, formal analysis, validation, writing – review and editing, project administration, resources. **Md. Jahirul Islam Mamun:** writing – original draft, visualization, methodology, software, formal analysis, data curation, writing – review and editing. **Mohi Uddin:** conceptualization, supervision, investigation, writing – review and editing, project administration, resources. **Masud Rana:** writing – review and editing. **Md. Ekramul Haque Ekram:** writing – original draft, formal analysis, software. **Khurshida Jahan Suma:** software, writing – original draft, formal analysis.

## Funding

The authors have nothing to report.

## Conflicts of Interest

The authors declare no conflicts of interest.

## Data Availability

Data will be made available from the corresponding author upon reasonable request.
